# ABO blood groups and nosocomial infection

**DOI:** 10.1017/S0950268823000432

**Published:** 2023-04-03

**Authors:** Xiao Zhong, Dong-Li Wang, Li-Hua Xiao, Lan-Fang Mo, Xiao-Feng Luo

**Affiliations:** 1 Infection Management Department, Shenzhen Hospital, University of Chinese Academy of Sciences, Shenzhen, China; 2 Testing Centre, Guangming District Centre for Disease Control and Prevention, Shenzhen, China

**Keywords:** ABO blood group, diagnosis related groups, nosocomial infection, nosocomial infection pathogen, nosocomial infection site, propensity score matching

## Abstract

The timely identification of the high-risk groups for nosocomial infections (NIs) plays a vital role in its prevention and control. Therefore, it is crucial to investigate whether the ABO blood group is a risk factor for NI. In this study, patients with NI and non-infection were matched by the propensity score matching method and a logistic regression model was used to analyse the matched datasets. The study found that patients with the B&AB blood group were susceptible to *Escherichia coli* (OR = 1.783, *p* = 0.039); the A blood group were susceptible to *Staphylococcus aureus* (OR = 2.539, *p* = 0.019) and *Pseudomonas aeruginosa* (OR = 5.724, *p* = 0.003); the A&AB blood group were susceptible to *Pseudomonas aeruginosa* (OR = 4.061, *p* = 0.008); the AB blood group were vulnerable to urinary tract infection (OR = 13.672, *p* = 0.019); the B blood group were susceptible to skin and soft tissue infection (OR = 2.418, *p* = 0.016); and the B&AB blood group were vulnerable to deep incision infection (OR = 4.243, *p* = 0.043). Summarily, the patient’s blood group is vital for identifying high-risk groups for NIs and developing targeted prevention and control measures for NIs.

## Background

More than 30 human blood groups have been discovered [1] to date, of which the ABO blood group, discovered by Landsteiner over a century ago [2], is the earliest and most familiar. ABO blood group antigens are widely distributed in platelets, epithelial cells, vascular endothelial cell membranes, extracellular fluid, and tissues [3]. Therefore, ABO blood group antigens are not pure blood group antigens; they are called histo-blood group antigens (HBGAs) [4]. Human ABO blood group antigens exhibit different phenotypes and genetically derived glycoconjugate structures located on the surface of red blood cells (RBCs) and play an active role in cell physiology and pathology [5, 6]. Glycoconjugate structures on RBCs serve various functions [7], including serving as receptors for bacteria and parasites [8, 9], foreign ligands, viruses, transporters, channels, structural proteins, adhesion molecules, enzymes, and structural proteins. ABO blood groups are statistically or physiologically associated with a variety of non-infectious diseases – such as acute kidney injury (AKI) [10], acute respiratory distress syndrome (ARDS) [11], myocardial disease [12], pancreatic cancer [13], embolism [14] – and infectious diseases [15, 16].

Nosocomial infections (NIs) are infections that patients acquire in hospitals [17]. Many pathogens cause NIs, including bacteria, viruses, fungi, parasites, and more. NI is an infectious disease type, and the patient’s ABO blood group may be a predisposing factor for NI, as it is for common community infections. However, whether the ABO blood group is a risk factor for NI and whether the ABO blood group is specific for different NI pathogens and different NI sites have rarely been reported in the literature.

This study used the propensity score matching (PSM) method to match patients with NIs caused by different NI pathogens and NI sites with non-infected patients. To balance the effects of patient disease severity, we accurately matched patients’ diagnosis-related groups (DRGs) with other common risk factors for NI. Multivariate regression analysis was performed using the matched data to determine the relationship between the ABO blood groups and NIs. This study’s results are important for identifying NI-susceptible and high-risk populations and for the precise prevention and control of NI.

## Methods

### Study object

The study was conducted at a 1350-bed grade III-A general hospital with 1,209 doctors and 1,312 nurses in Guangming District, Shenzhen, Guangdong, China. All hospitalised patients between January 2016 and July 2022 were selected as subjects.

### Inclusion and exclusion criteria

The case population included all patients with NIs during the study period, and the case population was stratified by NI pathogen and NI site. In the NI pathogen stratification, only patients infected with a single pathogen were included, and patients infected with multiple pathogens were excluded. Similarly, patients with simple infections were included in the NI site stratification, and patients with multi-site or multi-pathogen mixed infections were excluded. The control group included all hospitalised patients with no NIs during the study period and excluded those infected in the community. Patients with missing vital information such as demographic information, ABO blood group, DRGs, and invasive procedures in the case and control groups were also excluded. Even if the inclusion and exclusion criteria were met, the analysis would not be conducted if the number of NI pathogens and NI sites was too small.

### Diagnosis of NIs

NIs were diagnosed according to the Diagnostic Criteria for NIs (2001 edition) issued by the Ministry of Health of the People’s Republic of China [18]. First, the clinician reported the NIs to the infection management department through the Nosocomial Infection Information System (NIIS), and then two experienced infection control professionals would judge whether it was NI according to the standard. If there was a disagreement between the two professionals, a third professional would make the final decision. In addition, the NIIS also has a data interface with a Hospital Information System (HIS), Laboratory Information System (LIS), Picture Archiving and Communication System (PACS), Surgery and Anesthesia Information System (SAIS), and Medical record Management Information System (MMIS), which is capable of giving early warnings of NIs according to the patient’s body temperature, bacterial detection, inflammatory indicators, imaging diagnosis, use of antibiotics and other conditions. The two NI professionals would then judge whether the early warnings were NIs.

### DRGs of patients

DRGs are a vital tool for measuring the quality and efficiency of health care and medicare payments [19]. Essentially, DRG is a combined case classification scheme that divides patients into several diagnostic groups for management based on age, diagnosis, comorbidities, complications, treatment methods, disease severity, outcomes, resource consumption, and other factors. Therefore, patients in the same DRG have the same or similar disease severity. In the 1980s, research on controlling medical costs by case mix emerged internationally, with DRG as a representative scheme. China has also been actively exploring the reform of the payment method for DRG health insurance in recent years. In 2015, CN-DRG (2014 edition) was launched by the National Health and Family Planning Commission (NHFPC), which is based on China’s disease and surgery coding environment. In 2018, CN-DRG (2018 edition) was launched, with a total of 804 DRG groups. Using the 2018 version of the CN-DRG grouping device, our hospital’s medical record management department imported the information of all discharged patients in the previous month into the CN-DRG system at the beginning of each month, and the DRG and Relative Weight (RW) of patients were automatically obtained. Cases that did not meet the inclusion rules were classified into the uncertain group.

### Data collection procedure

Patient demographic information, NI information, microbiological test information, and diagnosis and treatment information were derived from the NIIS. The ABO blood group information was derived from the MMIS, which records blood group information for patients who have received or may require a blood transfusion. The DRG of patients was derived from the CN-DRG system.

### Study design

**Figure 1.**
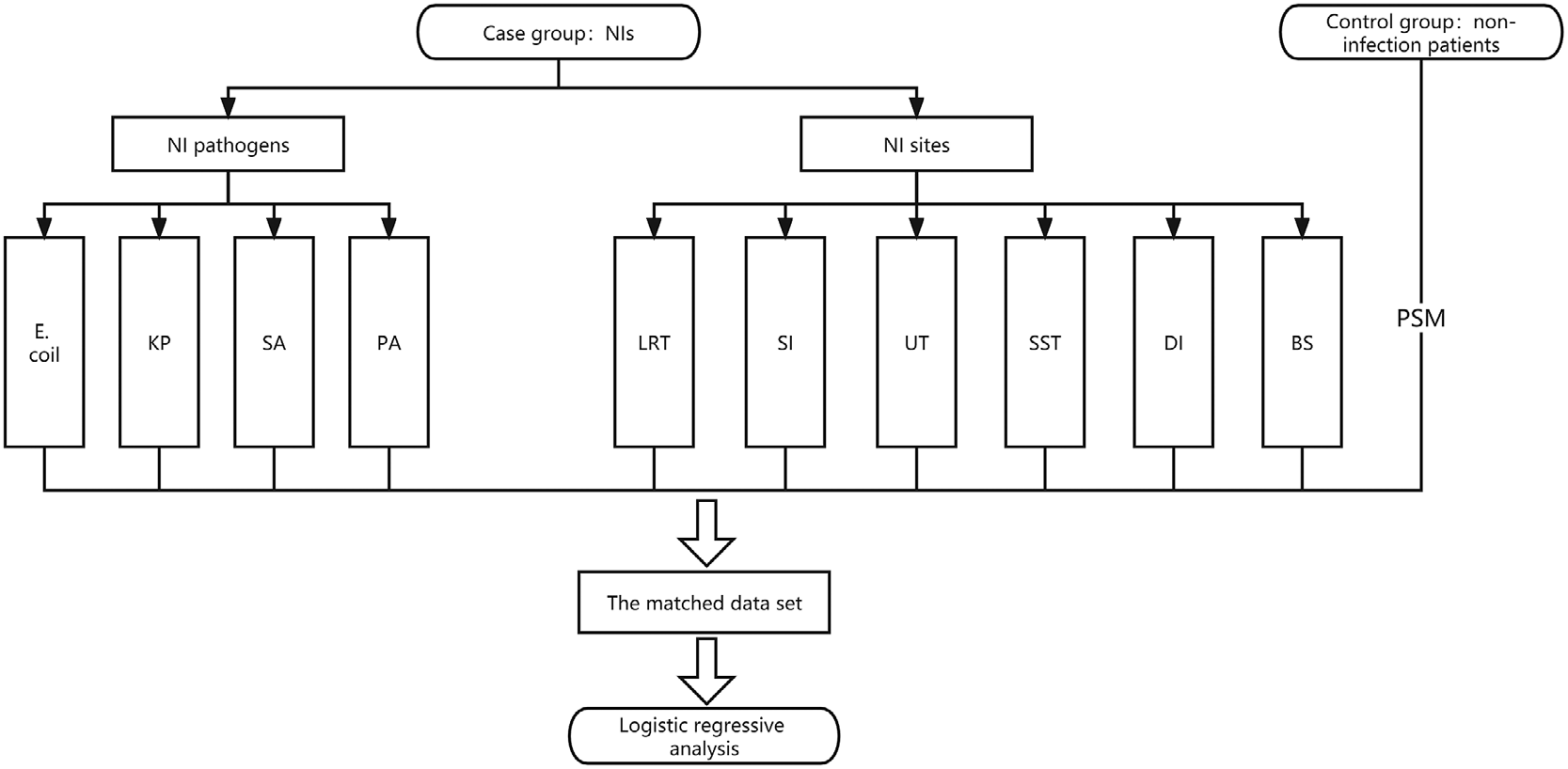
Study flow charts. NI pathogens and NI sites stratified the case groups. The stratified cases were matched with the control group by PSM. Finally, logistic regression was used to analyse the matched data. BS, blood system; DI, deep incision; *E. coli, Escherichia coli; KP, Klebsiella pneumoniae;* LRT, lower respiratory tract; NI, nosocomial infection; *PA, Pseudomonas aeruginosa*; PSM, propensity scores match; *SA, Staphylococcus aureus;* SI, superficial incision; SST, skin and soft tissue; UT, urinary tract.

A case–control study design was used in this study. The case group consisted of all NIs from January 2016 to July 2022 and was stratified by NI pathogen and NI site, while the control group consisted of inpatients with non-infectious diseases during the same period. The propensity scores for each group of patients were calculated using logistic regression. Based on the accurate matching of the DRG of the patients, a 1:1 case–control matching was performed based on the principle of nearest-neighbour matching, with a calliper value of 0.1. The matched covariates were gender, age, surgery, IMV, CVC, and IC. The matched dataset was used for logistic regression analysis. The covariables included the variables previously matched for propensity score, patients’ ABO blood group, and propensity score. The detailed study flow chart is shown in Figure 1.

### ABO blood group identification method

The microcolumn gel testing method was used to identify the patients’ blood group. First, 3–5 ml of venous blood was extracted from the patient, and the blood sample’s plasma and RBCs were separated. Then the isolated RBCs were configured with normal saline into a 5% RBC suspension. Finally, the RBC suspension was placed into the automatic blood group analyser (Changchun Boxun) for detection, and the detection results were read. The agglutinated RBCs located in the upper or middle part of the gel are judged as positive, while the agglutinated RBCs located in the bottom part are considered negative. If the agglutinated portion of the RBCs on the gel is not easily identifiable, it is judged as suspicious, and a manual determination is required.

### Methods for detection of NI pathogens

The VITEK2 bacterial identification and drug sensitivity analyser (Biomerieux, Merieux Alliance, France) was used to identify microorganisms. The identification of microorganisms followed the Clinical Laboratory Standards Institute (CLSI) recommendations.

### Statistic analysis

The data were input into Excel 365 to establish a database, and R software (version 4.2.1) was used to perform PSM and logistic regression analysis. Continuous variables conforming to normal distribution were described by mean (standard deviation), non-normal distribution data were described by median (Interquartile Range, IQR), and inter-group differences were compared by paired *t*-test or paired Wilcoxon rank sum test. Categorical variables were described by constituent ratio, and *χ*^2^ or Fisher’s exact test performed inter-group comparisons. All comparisons were statistically significant when *p* < 0.05.

## Results

### Baseline of cases and controls

**Figure 2.**
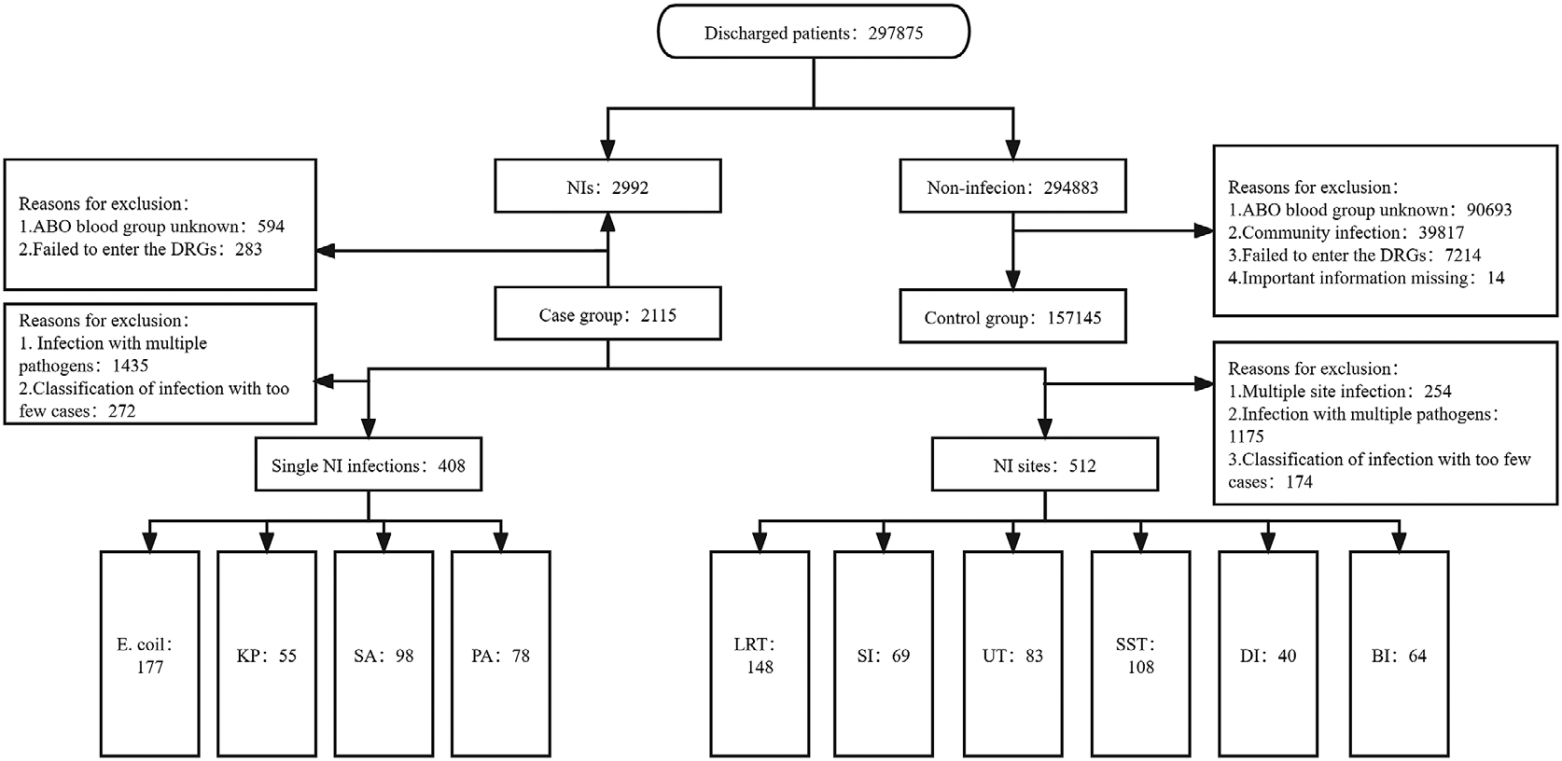
The inclusion and exclusion process of the case and control groups. BS, blood system; DI, deep incision; DRG, diagnosis-related groups; *E. coli, Escherichia coli; KP, Klebsiella pneumoniae;* LRT, lower respiratory tract; NI, nosocomial infection; *PA, Pseudomonas aeruginosa;* PSM, propensity scores match; *SA, Staphylococcus aureus;* SI, superficial incision; SST, skin and soft tissue; UT, urinary tract.

A total of 297,875 patients were discharged from the hospital between January 2016 and July 2022, including 2,992 patients with NIs and 294,883 without NIs. The inclusion and exclusion criteria excluded 594 patients without blood group information and 283 patients without DRG information from the case group. Meanwhile, the control group excluded 90,693 patients without ABO blood group information, 39,817 patients with community-acquired infections, 7,214 patients without DRG information, and 14 patients without vital information. Finally, the remaining 2,115 patients in the case group and 157,145 in the control group were enrolled in the study. The case population was stratified by NI pathogen and NI site. At the pathogen level of the NIs, 1,435 patients were excluded due to mixed infection with multiple pathogens, 272 patients were excluded from pathogen classification due to too few cases, and the remaining 408 patients were included in the study. At the NI sites level, 254 patients were excluded for multi-site mixed infection, 1,175 for multi-pathogen mixed infection, 174 for NI sites with too few cases, and the remaining 512 patients were included in the study, as shown in Figure 2. The final list of enrolled patients of cases and controls have been submitted as Supplementary Material available on the Cambridge Core website.

### Baseline after PSM

#### NI pathogen level

The propensity scores matched the patient groups stratified by NI pathogen and controls. The variables of gender, age, surgery, IMV, CVC, and IC were all balanced in each group after accurate matching of the DRGs of the patients, as shown in Table 1.

**Table 1.** Baseline after patient matching at NI pathogens level

Variable	All NIs	*E coli*	*KP*	*SA*	*PA*
Case group					
Matched no.	1,858	169	54	97	64
Unmatched no.	257	8	1	1	14
Control group					
Matched no.	1,858	169	54	97	64
Unmatched no.	136,207	64,650	22,575	57,869	24,498
Age (mean (SD))					
Case group	43.8(21.6)	39.8(19.4)	50.3(18.9)	33.9(23.3)	46.6(21.1)
Control group	43.0(20.6)	39.0(18.9)	53.1(19.7)	34.4(23.8)	48.8(20.4)
*p*-value	0.225	0.72	0.452	0.882	0.554
Female (%)					
Case group	753(40.5)	100(59.2)	21(38.9)	34(35.1)	14(21.9)
Control group	762(41.0)	97(57.4)	24(44.4)	33(34.0)	14(21.9)
*p*-value	0.789	0.825	0.696	1	1
Operation (%)					
Case group	1,064(57.3)	117(69.2)	25(46.3)	51(52.6)	40(62.5)
Control group	1,071(57.6)	119(70.4)	30(55.6)	53(54.6)	39(60.9)
*p*-value	0.842	0.906	0.441	0.886	1
IMV (%)					
Case group	279(15.0)	8(4.7)	14(25.9)	11(11.3)	21(32.8)
Control group	266(14.3)	8(4.7)	12(22.2)	10(10.3)	21(32.8)
*p*-value	0.578	1	0.822	1	1
CVC (%)					
Case group	423(22.8)	24(14.2)	23(42.6)	15(15.5)	22(34.4)
Control group	418(22.5)	22(13.0)	19(35.2)	14(14.4)	20(31.2)
*p*-value	0.875	0.874	0.554	1	0.851
IC (%)					
Case group	934(50.3)	101(59.8)	37(68.5)	30(30.9)	33(51.6)
Control group	925(49.8)	99(58.6)	34(63.0)	29(29.9)	28(43.8)
*p*-value	0.793	0.912	0.685	1	0.479

Abbreviations: CVC, central venous catheterization; *E. coli, Escherichia coli*; IC, indwelling catheter*;* IMV, invasive mechanical ventilation; *KP, Klebsiella pneumoniae;* NI, nosocomial infection; no., number; *PA, Pseudomonas aeruginosa*; *SA, Staphylococcus aureus;* SD, standard deviation.

#### NI site level

The propensity scores matched the patient groups stratified by NI sites and control. After the DRGs of the patients were precisely matched, the variables of gender, age, surgery, IMV, CVC, and IC were all balanced in each group, as shown in Table 2.

**Table 2.** Baseline after patient matching at NI sites level

Variable	Lower respiratory tract	Superficial incision	Urinary tract	Skin and soft tissue	Deep incision	Blood system
Case group						
Matched no.	121	69	74	107	39	59
Unmatched no.	27	0	9	1	1	5
Control group						
Matched no.	121	69	74	107	39	59
Unmatched no.	27,314	40,065	48,405	47,876	16,851	25,763
Age (mean (SD))						
Case group	49.30(22.41)	41.42(13.48)	49.72(18.42)	37.41(20.55)	42.67(14.89)	48.37(19.87)
Control group	49.12(21.80)	40.55(15.88)	48.20(18.91)	36.77(20.99)	41.21(13.17)	47.95(19.71)
*p*-value	0.949	0.729	0.623	0.821	0.647	0.908
Female (%)						
Case group	38(31.4)	21(30.4)	49(66.2)	25(23.4)	5(12.8)	35(59.3)
Control group	38(31.4)	23(33.3)	50(67.6)	27(25.2)	4(10.3)	38(64.4)
*p*-value	1	0.855	1	0.873	1	0.705
Operation (%)						
Case group	55(45.5)	59(85.5)	39(52.7)	74(69.2)	35(89.7)	19(32.2)
Control group	57(47.1)	60(87.0)	39(52.7)	74(69.2)	35(89.7)	20(33.9)
*p*-value	0.897	1	1	1	1	1
IMV (%)						
Case group	59(48.8)	1(1.4)	5(6.8)	3(2.8)	2(5.1)	4(6.8)
Control group	57(47.1)	1(1.4)	4(5.4)	2(1.9)	1(2.6)	2(3.4)
*p*-value	0.898	1	1	1	1	0.675
CVC (%)						
Case group	79(65.3)	7(10.1)	8(10.8)	7(6.5)	5(12.8)	10(16.9)
Control group	76(62.8)	8(11.6)	12(16.2)	7(6.5)	4(10.3)	10(16.9)
*p*-value	0.789	1	0.471	1	1	1
IC (%)						
Case group	81(66.9)	34(49.3)	57(77.0)	24(22.4)	15(38.5)	25(42.4)
Control group	84(69.4)	34(49.3)	56(75.7)	23(21.5)	14(35.9)	23(39.0)
*p*-value	0.783	1	1	1	1	0.851

Abbreviations: CVC, central venous catheterization; IC, indwelling catheter; IMV, invasive mechanical ventilation; NI, nosocomial infection; no., number; SD, standard deviation.

### Logistic regression results

#### NI pathogen level

The logistic regression analysis of all patients with NIs and without infection revealed that the ABO blood group was not a risk factor for NI. However, the stratified analysis of NI pathogens has shown that certain ABO blood groups have different susceptibilities to specific NI pathogens. For example, patients with the B&AB blood group were susceptible to *Escherichia coli* (OR = 1.783, *p* = 0.039); patients with the A blood group were susceptible to *Staphylococcus aureus* (OR = 2.539, *p* = 0.019) and *Pseudomonas aeruginosa* (OR = 5.724, *p* = 0.003); and patients with the A&AB blood group were susceptible to *P. aeruginosa* (OR = 4.061, *p* = 0.008), as shown in Table 3.

**Table 3.** Relationship between NI pathogens and ABO blood groups

NI pathogens	Blood group^a^	OR^b^	Lower 95% CI	Upper 95% CI	*p*-value
NIs	A	0.955	0.823	1.108	0.544
	B	1.098	0.942	1.281	0.232
	AB	1.099	0.846	1.427	0.479
	O	0.937	0.815	1.079	0.367
	A&AB	0.986	0.857	1.135	0.848
	B&AB	1.116	0.967	1.288	0.133
*E. coli*	A	0.660	0.366	1.191	0.168
	B	1.690	0.963	2.965	0.067
	AB	1.353	0.459	3.990	0.584
	O	0.802	0.465	1.383	0.428
	A&AB	0.758	0.441	1.304	0.317
	B&AB	1.783	1.029	3.091	0.039
*KP*	A	0.956	0.272	3.363	0.944
	B	1.250	0.309	5.057	0.754
	AB	4.084	0.636	26.218	0.138
	O	0.446	0.127	1.563	0.207
	A&AB	1.624	0.548	4.809	0.382
	B&AB	2.493	0.679	9.151	0.169
*SA*	A	2.539	1.164	5.540	0.019
	B	0.609	0.284	1.305	0.202
	AB	0.648	0.173	2.428	0.520
	O	0.773	0.372	1.604	0.489
	A&AB	2.111	0.992	4.493	0.053
	B&AB	0.565	0.275	1.162	0.121
*PA*	A	5.724	1.844	17.769	0.003
	B	0.485	0.159	1.477	0.203
	AB	0.486	0.057	4.110	0.507
	O	0.479	0.187	1.224	0.124
	A&AB	4.061	1.439	11.463	0.008
	B&AB	0.452	0.161	1.270	0.132

a The combination of A&AB or B&AB was analysed to study blood groups without A or B antibodies.

b Patients without a certain blood group were used as dummy variables. For example, the OR value of blood group A was compared with the dumb variable of the non-A blood group.

Abbreviations: CI, confidence interval; *E. coli, Escherichia coli; KP, Klebsiella pneumoniae;* NI, nosocomial infection; OR, odd ratio; *PA, Pseudomonas aeruginosa; SA, Staphylococcus aureus*.

#### NI site level

Patients with the AB blood group were more susceptible to urinary tract infections (OR = 13.672, *p* = 0.019). Patients with the B blood group were susceptible to skin and soft tissue infections (OR = 2.418, *p* = 0.016), while the A&AB blood group was a protective factor against the skin and soft tissue infections (OR = 0.462, *p* = 0.020). Patients with the B&AB blood group were susceptible to deep incision infection (OR = 4.243, *p* = 0.043), while the A blood group was a protective factor against deep incision infection (OR = 0.115, *p* = 0.011), as shown in Table 4.

**Table 4.** Relationship between NI sites and ABO blood groups

NI sites	Blood group^a^	OR^b^	Lower 95%CI	Upper 95%CI	*p*-value
Lower respiratory tract	A	1.452	0.752	2.803	0.266
	B	1.243	0.586	2.638	0.571
	AB	1.266	0.361	4.446	0.713
	O	0.554	0.287	1.069	0.078
	A&AB	1.492	0.795	2.799	0.213
	B&AB	1.273	0.650	2.492	0.482
Superficial incision	A	1.579	0.637	3.910	0.324
	B	0.779	0.298	2.037	0.610
	AB	2.759	0.602	12.643	0.191
	O	0.559	0.232	1.346	0.195
	A&AB	2.167	0.902	5.205	0.084
	B&AB	1.163	0.492	2.750	0.731
Urinary tract	A	1.399	0.540	3.621	0.490
	B	0.718	0.235	2.195	0.561
	AB	13.672	1.546	120.922	0.019
	O	0.527	0.217	1.280	0.157
	A&AB	2.394	0.969	5.915	0.058
	B&AB	1.633	0.577	4.621	0.355
Skin and soft tissue	A	0.608	0.309	1.196	0.149
	B	2.418	1.176	4.970	0.016
	AB	0.341	0.099	1.168	0.087
	O	1.041	0.552	1.965	0.901
	A&AB	0.462	0.241	0.886	0.020
	B&AB	1.492	0.791	2.813	0.216
Deep incision	A	0.115	0.021	0.615	0.011
	B	1.669	0.425	6.560	0.463
	AB	7.271	0.875	60.407	0.066
	O	1.254	0.347	4.524	0.730
	A&AB	0.525	0.149	1.852	0.317
	B&AB	4.243	1.049	17.166	0.043
Blood system	A	1.869	0.532	6.566	0.330
	B	1.670	0.573	4.866	0.347
	AB	0.446	0.060	3.334	0.431
	O	0.420	0.130	1.354	0.146
	A&AB	1.272	0.423	3.829	0.668
	B&AB	1.286	0.469	3.523	0.625

a The combination of A&AB or B&AB was analysed to study blood groups without A or B antibodies.

b Patients without a certain blood group were used as dummy variables. For example, the OR value of blood group A was compared with the dumb variable of the non-A blood group.

Abbreviations: CI, confidence interval; NI, nosocomial infection; OR, odd ratio.

## Discussion

Common risk factors for NIs include the age of the patient, as the elderly (>60 years of age) and infants (<1 year of age) are most vulnerable, the underlying disease of the patient and the severity of the disease, invasive procedures, IMV, CVC, IC, abuse of antibiotics, and use of immunosuppressants [17]. The underlying disease and its severity are important risk factors and confounding factors that must be addressed in case–control studies. This study used the international standard DRG to measure patients’ underlying disease status and severity. The DRG of the patients was accurately matched to remove its influence on the study and to ensure comparability between cases and the controls.

The ABO blood group has been associated with various infectious diseases, but whether it is a risk or predisposing factor for NIs is unknown. In this study, we first investigated the differences in the ABO blood group between all NI and non-infected patients. After balancing the baseline conditions of the two groups using PSM, performing logistic regression analysis found that the ABO blood group of the patients was not associated with NI occurrence. However, stratified matching according to NI pathogen and NI site showed that some blood types were strongly associated with specific NI pathogen and NI site.

The biological theories of blood group and infection are as follows [20]: First, the blood group antigens expressed by epithelial cells can act as receptors for the adhesion of bacteria, viruses, parasites, and other pathogens, resulting in different blood group antigens with different susceptibilities to pathogens [8]. Second, to avoid the immunological effects of blood group antibodies, some pathogens have features that mimic blood group antigens. For example, *meningococcal* bacteria produce either A or B antigens, depending on the environment, and contain an enzyme that converts A to B antigens. As a result, blood group A antibodies in patients with blood group B readily act as natural antibodies to the bacterial antigens, making patients with blood group B less susceptible to infection [21]. Third, blood group antigens can act as receptors for toxins and virulence factors produced by pathogens rather than directly binding to them [22]. For example, enterotoxins of *Vibrio cholerae* can bind to the antigens of the A and O blood groups [23].

This study found that B&AB blood group patients were susceptible to *E. coli* NI, which is consistent with the findings of Wittels and Lichtman [24], who conducted a comparative study of 115 patients with *E. coli* bloodstream infections and found that patients with B and AB blood groups had 1.6 times the risk of *E. coli* bloodstream infections (RR = 1.6, *p* = 0.01). Many studies have suggested that blood types B and AB are risk factors for *E. coli* infection [5, 25]. The reason for this may be that *E. coli* can mimic the properties of the B blood group antigens, making them susceptible to B blood group antibodies, whereas B and AB blood groups do not contain B antibodies, and therefore, patients with these blood groups are susceptible to *E. coli* infection [21, 26].

It has been reported that the surface of *S. aureus* has an A blood group antigen substance that can neutralise the A blood group antibodies in the B and O blood group [27] so that the A blood group without the A antibody is susceptible to *S. aureus* infection. Veselov and Malyshkina [28] tested 326 volunteers from four medical facilities and found that the A blood group accounted for most patients with *S. aureus.* Our study is consistent with the findings that patients with blood group A are a risk factor for *S. aureus* infections.

The study also found that patients with blood group A were susceptible to *P. aeruginosa*, consistent with literature reports [15]. Meanwhile, patients with the A&AB blood group are also susceptible to *P. aeruginosa*, as blood groups A and AB do not contain antibody A, so it is speculated that the biological principle may be related to the natural bacteriocidal effect of antibody A on *P. aeruginosa.* In addition, the adhesion of *P. aeruginosa* is mediated by a range of adhesion proteins, including PA-I and PA-II lectins. PA-I binds to d-galactose of erythrocyte glycosphingolipids and has the highest affinity for B and Pk antigens, while PA-II preferentially binds to H, A, and B antigens [29]. The study’s results also suggest that the affinity of the A antigen is higher than that of other blood group antigens.

The evolutionary pressure to change blood group types in response to infectious diseases has influenced the distribution of human blood types. For example, among the indigenous peoples of southwestern Africa and Central and South America, where malaria is endemic, the blood group O, which is resistant to malaria [30], accounts for the majority [9]. Blood group O contains both A and B antibodies, so it is less susceptible to bacteria than the non-O blood group [5, 31], which is one of the reasons for the extensive distribution of blood group O in China. However, this study found that the OR of the O blood group was lesser when compared to the non-O blood group, but not statistically significant. One of the reasons may be that the anti-A and anti-B in blood group O are IgG and have a lesser effect on complement activation when compared to the anti-B and anti-A (usually IgM) in blood groups A and B. In addition, the anti-B titre in the O blood group was lower than in the A blood group [32]. As a result, patients with blood group O have a protective effect against bacterial infections, but the protection is weak.

Different blood group antigens are susceptible to various sites of infection, and interactions between bacterial adhesins and host cell surface components determine tissue specificity for infection [33]. For example, Sulaiman et al. [34] studied 81 patients with recurrent catheter-associated urinary tract infections and showed that the risk of infection in patients with the non-O blood group was 3.57 times more than that of the O blood group (OR = 3.57, 95%CI = 1.05–12.5). Furthermore, different blood groups are specific to different infectious pathogens and a large number of blood group antigens are distributed on the surface of various human body tissues. Thus the tissue specificity is also directly related to the infectious pathogens. Studies have shown a significant increase in infections caused by *E. coli* and *Klebsiella pneumoniae* in patients with chronic upper urinary tract infections in the AB blood group [32]. In our study, the AB blood group was a risk factor for urinary tract infection, the B blood group was a risk factor for skin and soft tissue infection, and the B&AB blood group was a risk factor for deep incision infection. The reason may be that *E. coli* and *P. aeruginosa* account for the majority of these three types of infection, which is consistent with the study’s results based on pathogen stratification and has the effect of mutual validation. In addition, the A&AB blood group is a protective factor against skin and soft tissue infections, and the B&AB blood group is a protective factor against deep incision infections. The reason may be that the samples stratified by the NI site contain many different types of infectious bacteria, and the results may well reflect the mixing effects of these bacteria.

Although this study used the PSM to balance the baseline of the case and the control groups to remove the interference of confounding factors to the maximum extent, the study still had some shortcomings: First, the PSM matched a fraction of the main confounding factors. Due to data collection limitations, other confounding factors could not be matched, thus affecting the research results. Second, this study mitigates the impact on the patient’s disease by accurately matching the patient’s DRGs. However, due to the insufficient number of patients in some DRGs in the control group, some cases failed to be matched, resulting in a loss of cases and thus affecting the reliability of the findings. Finally, the study is retrospective and may have a recall bias. In addition, the sample was obtained from a Level III-A general hospital in the region and was a single-centre study, which may also have affected the extrapolation of the results.

## Conclusions

Summarily, this study explored the differences in the distribution of ABO blood groups between NI patients and non-infected patients through PSM and accurately matched the DRGs of the patients to eliminate the influence of disease severity to the greatest extent. Several blood groups were found to be statistically specific for *E. coli* infection, *S. aureus* infection, *P. aeruginosa* infection, urinary tract infection, skin soft tissue infection, and deep incision infection. Although a patient’s blood group is an intrinsic factor of the patient, like gender and age, and cannot be changed, we can classify these groups as high-risk groups for NI. Measures such as personal protection, disinfection, and hand hygiene should be taken during medical services to protect patients from NIs. Although the ABO blood group is statistically associated with NI, its biological mechanism has not been fully elucidated, and further studies of the ABO blood group and NI at the molecular level are needed in the future.

## Data Availability

The data used in the study were available from the Infection Management Department of the Shenzhen Hospital of the University of Chinese Academy of Sciences.

## Supplementary material

For supplementary material accompanying this paper visit http://doi.org/10.1017/S0950268823000432. click here to view supplementary material
